# The design and synthesis of an antibacterial phenothiazine–siderophore conjugate

**DOI:** 10.3762/bjoc.14.242

**Published:** 2018-10-16

**Authors:** Abed Tarapdar, James K S Norris, Oliver Sampson, Galina Mukamolova, James T Hodgkinson

**Affiliations:** 1 Leicester Institute of Structural and Chemical Biology, and Department of Chemistry, University of Leicester, George Porter Building, University Road, Leicester, LE1 7RH, UK; 2 Leicester Tuberculosis Research Group, Department of Infection, Immunity and Inflammation, University of Leicester, Maurice Shock Medical Sciences Building, University Road, Leicester, LE1 9HN, UK

**Keywords:** NDH-2, phenothiazine, siderophore, siderophore–antibiotic, siderophore conjugate

## Abstract

Siderophore–antibiotic conjugates consist of an antibiotic covalently linked by a tether to a siderophore. Such conjugates can demonstrate enhanced uptake and internalisation to the bacterial cell resulting in significantly reduced MIC values and extended spectrum of activity. Phenothiazines are a class of small molecules that have been identified as a potential treatment for multidrug resistant tuberculosis and latent TB. Herein we report the design and synthesis of the first phenothiazine–siderophore conjugate. A convergent synthetic route was developed whereby the functionalised phenothiazine component was prepared in four steps and the siderophore component also prepared in four steps. In *M. smegmatis* the functionalised phenothiazine demonstrated an equipotent MIC value in direct comparison to the parent phenothiazine from which it was derived. The final conjugate was synthesised by amide bond formation between the two components and global deprotection of the PMB protecting groups to unmask the catechol iron chelating groups of the siderophore. The synthesis is readily amenable to the preparation of analogues whereby the siderophore component of the conjugate can be modified. The route will be used to prepare a library of siderophore–phenothiazine conjugates for full biological evaluation of much needed new antibacterial agents.

## Introduction

One of the biggest challenges facing the modern society is antibiotic resistance and the prospect of current antibiotics becoming near redundant against previously treatable infections [1]. To meet this challenge there is a desperate need for new antibiotics, antibiotic targets and strategies to enhance the efficacy of current antibiotics [2]. One novel strategy which is receiving significant interest is the manipulation of bacterial iron transport pathways to deliver antibiotics to the bacterial cell [3]. Iron is essential for bacterial survival and bacteria secrete high affinity iron chelating molecules to scavenge and solubilise Fe^3+^ from the extracellular environment [3]. The siderophore–Fe complex is recognised by specific receptor proteins on the outer membrane of the bacteria and internalised into the bacterium cell by active transport [4].

Siderophore–antibiotic conjugates consist of an antibiotic covalently linked by a ‘tether' to a siderophore. Such conjugates overcome the bacterial membrane permeability barrier and facilitate active transport of the antibiotic to its internal target. Siderophore–antibiotic conjugates can demonstrate significantly enhanced bacterial killing potencies and an extended spectrum of activity [5–6]. Although there has been success reported with a number of antibiotics with differing targets the most success to date has been achieved with beta-lactam-based siderophore conjugates targeting membrane associated penicillin binding proteins (PBPs) [7]. Cefiderocol (S-649266) is a beta-lactam–siderophore conjugate currently in phase III clinical trials which demonstrates enhanced potency against Gram-negative bacteria including multidrug resistant (MDR) Gram-negative pathogens [8]. One hypothesis for the success of siderophore conjugates targeting PBPs, in comparison to other antibiotic targets, is that PBPs are membrane associated and it is not necessary for the siderophore conjugate to cross into the bacterial cytoplasm [7].

**Figure 1:**
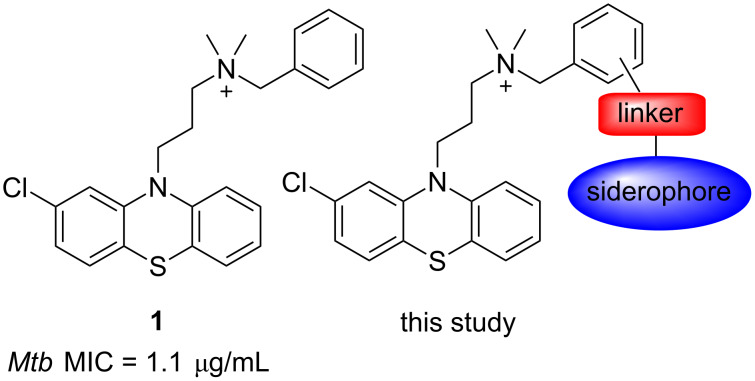
NDH-2 is a validated target for **1** with an MIC of 1.1 µg/mL against *M. tuberculosis*.

Phenothiazines are a privileged scaffold in drug discovery most noted for their use as antipsychotic drugs including chlorpromazine, trifluoperazine, and thioridazine. However, such drugs have also long been noted for their significant antimicrobial activity particularly against *Staphylococcus aureus* and *Mycobacterium tuberculosis* [9–10]. The emergence of MDR-TB has led to structure–activity studies to enhance the antitubercular activity of phenothiazines leading to the identification of chlorpromazine analogue **1** (Figure 1) which demonstrates MIC values comparable to first-line TB drugs in vitro [11]. However, the potency of such phenothiazines, including **1**, needs to be significantly increased to have more activity in vivo and direct clinical application [11]. A validated target of **1** has been identified as type II NADH dehydrogenase (NDH-2), a respiratory enzyme essential for growth in *M. tuberculosis* and other bacterial species [11]. NDH-2 is absent in mammalian cells and similar to PBPs is associated with the bacterial membrane [12]. Considering the significant antibacterial activity of phenothiazines, in particular the anti-TB activity of **1**, and their membrane-associated NDH-2 target we hypothesised **1** may be an interesting candidate for siderophore conjugation.

## Results and Discussion

Typically siderophore–antibiotic conjugates consist of a linker joining the siderophore and antibiotic components. As the target is membrane-associated NDH-2 we decided to functionalise our conjugate with a non-cleavable linker. A polyethylene glycol (PEG) linker was selected as PEG linkers demonstrate enhanced water solubility in comparison to alkyl chain linkers. We then had to make a decision on the position of attachment for the PEG linker to compound **1**. For siderophore conjugates, it is crucial that the linker is attached to a position in **1** such that the antibacterial activity is not compromised. Based on previous structure–activity studies of **1** by Bate et al., whereby a methoxy group was positioned on the *para*-position of the phenyl ring of **1** without loss of activity, we hypothesised this may be a suitable position for PEG linker attachment [13].

**Scheme 1:**
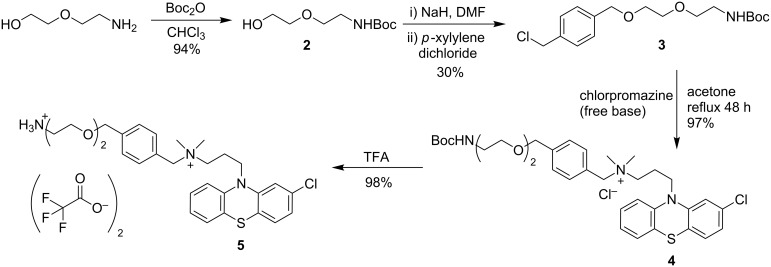
Synthesis of phenothiazine-PEG-amine component.

From commercially available 2-(2-aminoethoxy)ethanol the amine functionality was Boc-protected under standard conditions to give compound **2** (Scheme 1). Under basic conditions **2** underwent an S_N_2 reaction with commercially available *p*-xylylene dichloride to give **3**. Complete conversion of starting material was observed by ^1^H NMR, however, the isolated yield of **3** was poor possibly due to competing N-alkylation of the Boc group. Isolation of the O-alkyl product **3** was confirmed by ^13^C NMR. Despite the poor isolated yield of **3** the mass recovery was more than suitable to progress to the next steps. Initially, the reaction of **3** with chlorpromazine (free base) was attempted at room temperature; however, it was found refluxing was required to drive the reaction to completion to generate compound **4** in excellent yield. The final PEG-amine-functionalised phenothiazine **5** was isolated after removal of the Boc protecting group in TFA. Initial attempts at aqueous work-up conditions to isolate the free base resulted in lower isolated yields of **5** due to its high water solubility, and it was decided **5** would be progressed further as the TFA salt avoiding aqueous work-up.

To determine if the antibacterial activity of the derivatised phenothiazine was retained the MIC of compound **4** was determined in direct comparison to synthesised **1** against *Mycobacterium smegmatis* (see Supporting Information File 1). *M. smegmatis* is commonly used as a first assessment for antituberculosis activity. We were pleased to observe side by side compound **4** exhibited equimolar MIC values to **1** (6.25 μM, **1** and **4**) against *M. smegmatis*.

Next our attention turned to the siderophore component of the conjugate. In our proof of concept study we chose to synthesise the bis-catechol siderophore azotochelin. Catechol-based siderophores can act as xenosiderophores and be recognised for uptake by Gram-negative bacteria and *mycobacteria* [14–15]*.* Most commonly benzyl protecting groups are used in the synthesis of catechol siderophores and cleaved in the final step by palladium catalysed hydrogenation. However, as our final conjugate contains an aromatic halide we wanted to avoid hydrogenation as the final step and we instead chose to use the *para*-methoxybenzyl (PMB) protecting group which can be removed under acidic conditions.

**Scheme 2:**
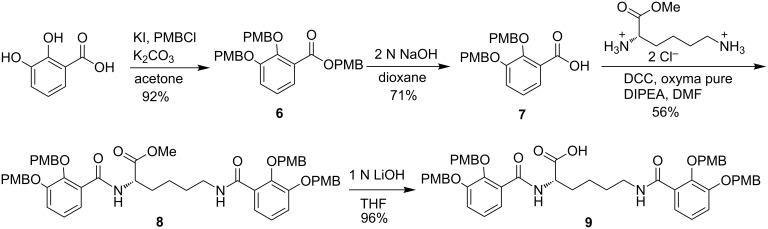
Synthesis of the azotochelin siderophore component.

PMB-protected benzoic acid building block **7** was prepared following a literature procedure in two steps (Scheme 2) [16]. Building block **7** was coupled to commercially available L-lysine methyl ester dihydrochloride to yield **8** in moderate yield. The majority of the dicyclohexylurea byproduct could be removed by cooling a solution of the residue dissolved in acetonitrile; however, column chromatography was required for analytical pure material. Ester hydrolysis proceeded smoothly in excellent yield to give the protected siderophore component **9**.

**Scheme 3:**
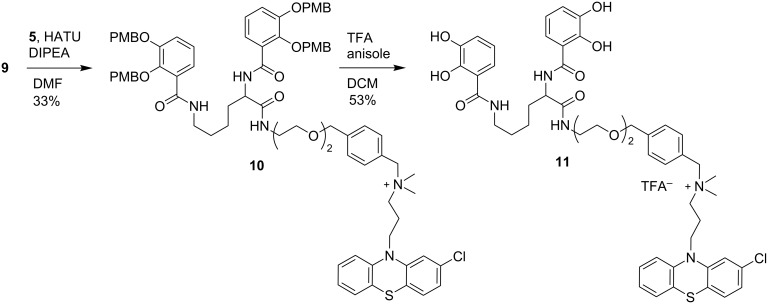
Final conjugation and deprotection to yield a phenothiazine siderophore conjugate.

Finally the phenothiazine component **5** and siderophore component **9** where coupled together by amide bond formation using HATU (Scheme 3). Although the isolated yield of **10** is poor the reaction proceeded with good conversion to the desired product; however, on purification an unknown contaminant was challenging to separate from **9** and we wanted to progress with only analytically pure material for the final deprotection step. We were also surprised to observe compound **10** had undergone racemisation under these conditions. The exact cause of racemisation is unknown, but may possibly be due to the four equivalents of DIPEA used to ensure **5** is converted to its free base. This will be investigated further for the synthesis of future conjugates. In the final PMB global deprotection step we were pleased to observe the formation of our desired final phenothiazine–sideophore conjugate in moderate yield. The addition of anisole to the reaction mixture was found to be essential to inhibit competing electrophilic substitution side reactions. A number of techniques where investigated for purification including standard chromatography, recrystallization and trituration as the crude ^1^H NMR revealed the majority of the desired product. However, purification by semi-preparative HPLC was required to obtain analytically pure material for biological evaluation.

## Conclusion

In conclusion we have developed a novel synthetic route to the first phenothiazine–siderophore conjugate. This was achieved by a convergent two component synthesis in a total of ten synthetic steps. The work extends research into antibacterial phenothiazines and siderophore-mediated antibiotic delivery. A library of mono-, bis- and tris-catechol phenothiazine–siderophore conjugates are currently being prepared using this route. Their synthesis and MIC values against pathogenic mycobacteria, Gram-negative bacteria and Gram-positive bacteria, along with compound **11**, will be reported in due course.

## Supporting Information

File 1 Full experiential protocols, characterisation of compounds including ^1^H and ^13^C NMR spectra, and biological evaluation of compound **4**.
