# Supplementary material for: The design and synthesis of an antibacterial phenothiazine–siderophore conjugate
Source: Beilstein J Org Chem. 2018 Oct 16;14:2646–50. doi: 10.3762/bjoc.14.242 (PMC6204836; doi:10.3762/bjoc.14.242)
Supplement: File 1 — Full experiential protocols, characterisation of compounds including 1H and 13C NMR spectra, and biological evaluation of compound 4. [file Beilstein_J_Org_Chem-14-2646-s001.pdf]

Supporting Information

for

The design and synthesis of an antibacterial phenothiazine–siderophore conjugate

Abed Tarapdar[1], James K.S. Norris[1], Oliver Sampson[2], Galina Mukamolova[2] and James T. Hodgkinson*[1]

Address: [1]Leicester Institute of Structural and Chemical Biology, and Department of Chemistry, University of Leicester, George Porter Building, University Road, Leicester, LE1 7RH, UK and [2]Leicester Tuberculosis Research Group, Department of Infection, Immunity and Inflammation, University of Leicester, Maurice Shock Medical Sciences Building, University Road, Leicester, LE1 9HN, UK

Email: James T. Hodgkinson - JTHodgkinson@le.ac.uk

* Corresponding author

Full experiential protocols, characterisation of compounds including ^{1}H and ^{13}C NMR spectra, and biological evaluation of compound 4

Contents

General information……………………………………………………………………...S2

Experimental…………………………………………………………………………...S3

Biological evaluation…………………………………………………………………S14

References……………………………………………………………………………..S15

Novel spectra…………………………………………………………………………..S16

General information

All reagents and solvents were obtained from Sigma Aldrich, Acros Organics, Fisher Scientific and were used as supplied unless stated otherwise. Glassware was dried in oven at 100 °C for 12 hours for moisture sensitive reactions. Unless otherwise stated reactions were performed under nitrogen using anhydrous solvents. Room temperature refers to ambient temperature. Temperatures of 0 °C were maintained using an ice-water bath. The reactions were monitored by thin-layer chromatography (TLC) on aluminium backed silica gel. Unless otherwise stated flash column chromatography was carried out with Silica Gel 60 using commercial solvents. All evaporations in vacuo were performed under reduced pressure using a Büchi rotary evaporator.

NMR spectroscopy (^{1}H and ^{13}C) were recorded on Bruker AV400 and AV500 machines in deuterated solvents, $CDCl_3$, MeOD. The machine and solvent used is specified in the data. ^{1}H NMR Chemical shifts (δ) are quoted in parts per million (ppm) and quoted to the nearest 0.01 ppm with tetramethylsilane as a reference. Coupling constants (*J)* are calculated in Hertz (Hz) to the nearest 0.1 Hz and found using ACD Labs/Spectrus Processor 2016.1.1 (File Version S50S41). NMR data are reported as: chemical shift, integration, multiplicity [b, broad; s, singlet; d, doublet; t, triplet; q, quartet; quin, quintet; sept, septet; m, multiplet; or as a combination (e.g., dd, dt, etc.)] and coupling constant(s). ^{13}C NMR spectra were recorded by broadband proton decoupling. ^{13}C NMR chemical shifts (δ) are quoted to the nearest 0.1 ppm and referenced to the residual non-deuterated solvent peak. Were C, CH, CH_2 and CH_3 have been defined in ^{13}C NMR assignments this has been determined by DEPT.

Mass spectrometry was carried out on a Micromass Quatro LC electrospray spectrometer. High resolution mass spectrometry (HRMS) was carried out on a Waters Acquity XEVO Q Time of flight spectrometer with either electrospray (ES+) or Atmospheric Solids Analysis Probe (ASAP) ionisation. Solutions were either made up in HPLC Grade acetonitrile (MeCN) and deionised water (1:1) or HPLC grade methanol. IR Spectroscopy was carried out using a Bruker Alpha, Platinum ATR with diamond wafer window. Absorptions are reported in wavenumbers (cm^{-1}) and classified as m medium; s strong; br broad. The Optical rotation of chiral compounds were determined using a Perkin Elmer polarimeter 341 at ambient temperature, wavelength 589 nm, *c* g/100 mL.

Experimental

N-Benzyl-3-(2-chloro-10H-phenothiazin-10-yl)-N,N-dimethylpropan-1-aminium bromide (**1**)

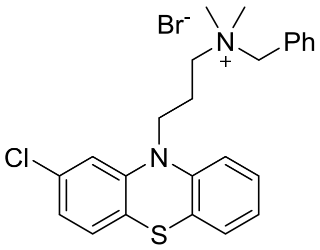

Chlorpromazine hydrochloride was free based by dissolving chlorpromazine hydrochloride (0.5 g, 1.57 mmol) in water (10 mL) and adding solid K_2CO_3 until pH 10. The aqueous phase was then extracted with chloroform (5 × 5 mL). The organic phases where combined, dried over anhydrous Na_2SO_4, filtered under gravity and the organic solvent removed in vacuo. The chlorpromazine free base (0.49 g, 1.55 mmol) was then dissolved in acetone (5 mL) and combined with benzyl bromide (0.16 mL, 1.41 mmol) in acetone (5 mL), and the reaction mixture was stirred at room temperature for 18 h. Diethyl ether was added and the reaction mixture and left to cool at 0 ^{o}C until a white precipitate formed. The precipitate was collected, triturated with cold diethyl ether (3 × 3 mL) and dried *in vacuo* to afford compound **1** as a white solid (0.49 g, 1.00 mmol, 65 %).

^{1}H NMR (400 MHz, CHLOROFORM-*d*) δ ppm 7.43 - 7.48 (2 H, m), 7.33 - 7.39 (2 H, m), 7.23 (1 H, d, *J*=6.5 Hz), 7.24 (1 H, td, *J*=8.0, 1.6 Hz), 7.16 (1 H, dd, *J*=7.6, 1.6 Hz), 7.06 (1 H, d, *J*=8.2 Hz), 7.00 (1 H, td, *J*=7.6, 1.2 Hz), 6.96 (2 H, dd, *J*=8.2, 1.8 Hz), 6.89 (1 H, d, *J*=2.0 Hz), 4.85 (2 H, s), 4.08 (2 H, t, *J*=5.9 Hz), 3.69 - 3.75 (2 H, m), 3.14 (6 H, s), 2.31 - 2.40 (2 H, m)

^{13}C NMR (101 MHz, CHLOROFORM-*d*) δ ppm 146.1, 143.6, 133.7, 133.0 (2 x CH), 130.6, 129.2 (2 x CH), 128.2, 128.0, 127.8, 127.1, 125.8, 124.6, 123.8, 123.2, 116.7, 116.5, 67.8, 61.3, 49.9 (2 x CH_3), 44.1, 20.5

m/z (ESI) found 409 (^{35}Cl), 411 (^{37}Cl) $[M]^+$

Spectroscopic data consistent with literature[1]

tert-Butyl (2-(2-hydroxyethoxy)ethyl)carbamate (**2**)

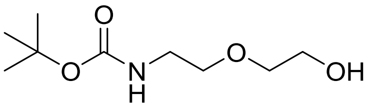

To 2-(2-aminoethoxy)ethanol (1.05 g, 10.0 mmol) in chloroform (10 mL) was added di-*tert*-butyl dicarbonate (2.19 g, 10 mmol) portionwise at 0 °C. The reaction mixture was left to stir vigorously at room temperature for 1.5 h. Water (30 mL) was added and the two phases where separated. The organic phase was collected and the aqueous phase was extracted further with chloroform (2 × 30 mL). The organic phases were combined, dried over anhydrous $MgSO_4$, filtered under gravity and the organic solvent removed in vacuo to give compound **2** as a pale yellow oil (1.93 g, 9.40 mmol, 94%).

^{1}H NMR (400 MHz, CHLOROFORM-*d*) δ ppm 4.96 (1H, br s), 3.70–3.78 (2H, m), 3.52–3.62 (4H, m), 3.28–3.39 (2H, m), 2.26 (1H, br t, J=5.9 Hz), 1.45 (9H, s)

^{13}C NMR (100 MHz, CHLOROFORM-*d*) δ ppm 156.2 (C), 79.2 (CH_2), 72.3 (CH_2), 70.2 (CH_2). 61.5 (CH_2), 40.3 (CH_2), 28.4 (3 x CH_3);

m/z (ESI) 228 $[M+Na]^+$;

HRMS (ES+) $C_9H_{19}NO_4Na$ $[M+Na]^+$ requires 228.1212 found 228.1214.

Spectroscopic data consistent with literature[2]

tert-Butyl (2-(2-((4-(chloromethyl)benzyl)oxy)ethoxy)ethyl)carbamate (**3**)

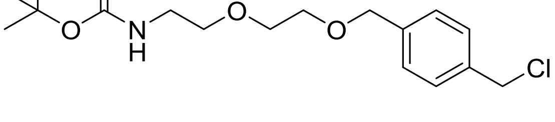

To **2** (1.00 g, 4.8 mmol) in DMF (10 mL) was added sodium hydride, 60% in mineral oil, (0.20 g, 5.3 mmol) portionwise at 0 °C and the reaction mixture left to stir for 10 min. α,α′-Dichloro-*p*-xylene (0.85 g, 4.8 mmol) was then added to the reaction mixture and left to stir at room temperature for 20 h. The reaction mixture was quenched with the addition of water (50 mL). The aqueous phase was then extracted with diethyl ether (3 × 35 mL). The yellow precipitate present on extraction was removed by filtration under gravity. The organic phases where combined and washed with water (40 mL) and LiCl (5 % aqueous solution, 2 x 40 ml). The organic phase was dried over anhydrous Mg_2SO_4, filtered under gravity and the organic solvent removed in vacuo to give a pale yellow oil (1.2 g). The remaining oil was purified by flash chromatography on silica by gradient elution (10–30 % ethyl acetate in petroleum ether (40–60)) to yield compound **3** as a colourless oil (0.50 g, 1.45 mmol, 30%).

^{1}H NMR (400 MHz, CHLOROFORM-*d*) δ ppm 7.28 - 7.45 (4 H, m), 4.97 (1 H, br s), 4.59 (2 H, s), 4.57 (2 H, s), 3.59 - 3.66 (4 H, m), 3.54 (2 H, t, *J*=5.1 Hz), 3.31 (2 H, br q, *J*=5.1 Hz) 1.44 (9 H, s)

^{13}C NMR (101 MHz, CHLOROFORM-*d*) δ ppm 156.0 (C), 138.5 (C), 136.9 (C), 128.7 (2 x CH), 128.0 (2 x CH), 79.2 (C), 72.8 (CH_2), 70.3 (CH_2), 70.2 (CH_2), 69.4 (CH_2), 46.0 (CH_2), 40.4 (CH_2), 28.4 (3 x CH_3)

HRMS (ES+) $C_{17}H_{26}NO_4Na\ ^{35}Cl$ $[M+Na]^+$ requires 366.1448 found 366.1458; $C_{17}H_{26}NO_4Na\ ^{37}Cl$ $[M+Na]^+$ requires 368.1419 found 368.1445

IR: *ν* (cm^{-1}) (nujol) = 3250 (m, amide N-H), 1720 (s, C=O)

3-(2-Chloro-10H-phenothiazin-10-yl)-N-(4-(11,11-dimethyl-9-oxo-2,5,10-trioxa-8-azadodecyl)benzyl)-N,N-dimethylpropan-1-aminium chloride (**4**)

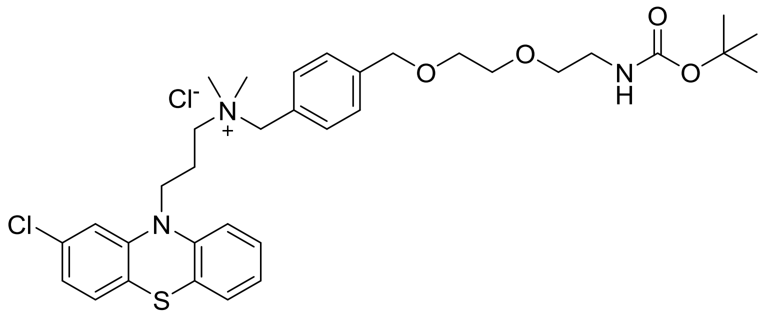

The free base chlorpromazine (0.37 g, 1.31 mmol), see preparation of compound **1**, was dissolved in acetone (3 mL) and combined with **3** (0.40 g, 1.16 mmol) dissolved in acetone (3 mL). The reaction mixture was heated at reflux with stirring for 24 h and at 40 °C for a further 24 h. The acetone was removed in vacuo to yield **4** as an orange amorphous solid (0.74 g, 1.12 mmol, 97%).

^{1}H NMR (400 MHz, CHLOROFORM-*d*) δ ppm 7.36 (2 H, d, *J*=8.2 Hz) 7.25 (2 H, d, *J*=8.0 Hz) 7.16 (1 H, td, *J*=7.4, 1.6 Hz) 7.08 (1 H, dd, *J*=7.6, 1.4 Hz) 6.99 (1 H, d, *J*=8.0 Hz) 6.85 - 6.94 (3 H, m,) 6.81 (1 H, d, *J*=1.8 Hz) 4.91 (1 H, br s) 4.78 (2 H, s) 4.50 (2 H, s) 4.00 (2 H, t, *J*=5.9 Hz) 3.55 - 3.66 (6 H, m) 3.49 (2 H, t, *J*=5.3 Hz) 3.26 (2 H, br q, *J*=5.1 Hz) 3.08 (6 H, s) 2.26 - 2.32 (2 H, m) 1.35 (9 H, s,)

^{13}C NMR (126 MHz, CHLOROFORM-*d*) δ ppm 155.9, 146.2, 143.5, 140.7, 133.5, 132.9 (2 x CH), 128.1 (2 x CH), 128.0 (2 x CH), 127.7, 126.4, 125.5, 124.3, 123.6, 122.9, 116.9, 116.5, 79.2, 72.4, 70.3 (2 x CH_2), 69.7, 67.4, 60.8, 50.3 (2 x CH_3), 44.0, 40.3, 28.4 (3 x CH_3), 20.38

HRMS (ES+) $C_{34}H_{45}N_3O_4S$ ^{35}Cl $[M]^+$ requires 626.2819 found 626.2839; $C_{34}H_{45}N_3O_4S$ ^{37}Cl $[M]^+$ requires 628.2790 found 628.2773

IR: *ν* (cm^{-1}) (nujol) = 3250 (m, amide N-H), 1722 (s, C=O)

N-(4-((2-(2-Ammonioethoxy)ethoxy)methyl)benzyl)-3-(2-chloro-10H-phenothiazin-10-yl)-N,N-dimethylpropan-1-aminium di(trifluoroacetate) (**5**)

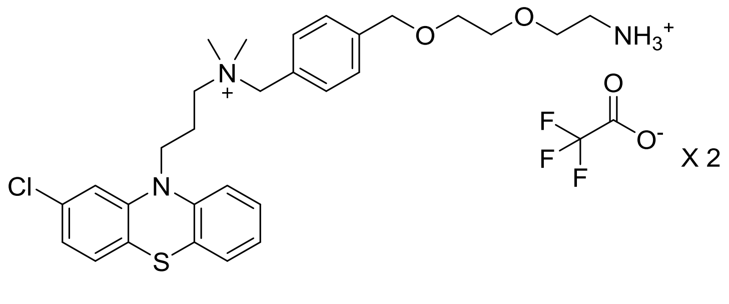

To **4** (0.45 g, 0.68 mmol) dissolved in DCM (4 mL) at 0 °C was added TFA (2 mL). The reaction mixture was left to stir for 15 h at room temperature. A flow of nitrogen was used to evaporate the solvent from the reaction mixture, which was then dissolved in DCM (5 mL) and removed in vacuo, this was repeated four times to removed residual TFA. The remaining

gum was triturated with diethyl ether (3 × 5 mL) and the residual solvents removed *in vacuo* to yield compound **5** as a light brown gum (0.50 g, 0.66 mmol, 98%).

^{1}H NMR (400 MHz, METHANOL-d_4) δ ppm 7.32 (2 H, d, *J*=8.3 Hz), 7.26 (2 H, d, *J*=8.3 Hz), 7.17 - 7.22 (1 H, m), 7.09 (1 H, dd, *J*=7.6, 1.5 Hz), 7.00 - 7.06 (3 H, m), 6.91 - 6.97 (2 H, m), 4.55 (2 H, s,), 4.36 (2 H, s), 4.05 (2 H, t, *J*=5.9 Hz), 3.59 - 3.70 (6 H, m), 3.24 - 3.33 (2 H, m), 3.06 (2 H, t, *J*=5.6 Hz), 2.87 (6 H, s), 2.15 - 2.27 (2 H, m)

^{13}C NMR (101 MHz, CHLOROFORM-*d*) δ ppm 146.1 (C), 143.5 (C), 141.4 (C), 133.8 (C), 132.9 (2 x CH), 128.3 (CH), 128.2 (2 x CH), 128.0 (CH), 127.9 (CH), 126.0 (C), 125.8 (C), 124.5 (C), 123.8 (CH), 123.2 (CH), 116.6 (CH), 116.5 (CH), 72.9 (CH_2), 72.4 (CH_2), 70.3 (CH_2), 70.0 (CH_2), 68.0 (CH_2), 61.5 (CH_2), 49.7 (2 x CH_3), 43.9 (CH_2), 41.5 (CH_2), 20.4 (CH_2)

HRMS (ES+) $C_{29}H_{37}N_3O_2S$ ^{35}Cl $[M]^+$ requires 526.2295 found 526.1458

IR: *ν* (cm^{-1}) (nujol) = 3250 (m, amine N-H), 1560 (m, aromatic), 1550 (m, aromatic), 1525 (m, aromatic).

4-Methoxybenzyl 2,3-bis((4-methoxybenzyl)oxy)benzoate (**6**)

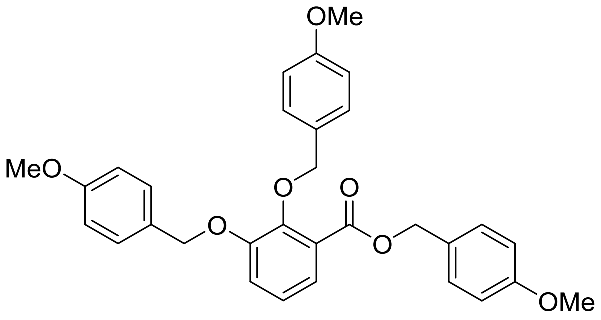

A white slurry of 2,3-dihydroxybenzoic acid (0.30 g, 1.94 mmol), 4-methoxybenzyl chloride (0.85 mL, 6.23 mmol), KI (1.03 g, 6.23 mmol), K_2CO_3 (1.87 g, 13.56 mmol) in acetone (14.3 mL) was stirred at reflux for 3 days. The mixture was cooled to room temperature and solvent removed in vacuo. The remaining solid was dissolved in water (20 mL) and extracted with dichloromethane (3 × 25 mL). The combined organic layers were dried over anhydrous Na_2SO_4, filtered and solvent removed in vacuo to yield compound 6 as a brown solid (0.92 g, 1.80 mmol, 92%).

^{1}**H NMR** (500 MHz, CHLOROFORM-*d*) δ ppm 7.24 - 7.30 (4 H, m), 7.16 - 7.23 (1 H, m), 7.11 (2 H, d, *J*=8.3 Hz), 7.03 (1 H, dd, *J*=8.1, 1.4 Hz), 6.96 (1 H, t, *J*=7.9 Hz), 6.73 - 6.83 (4 H, m), 6.68 (2 H, d, *J*=8.5 Hz), 5.17 (2 H, s), 4.96 (2 H, s), 4.88 (2 H, s), 3.74 (3 H, s), 3.72 (3 H, s), 3.70 (3 H, s);

^{13}**C NMR** (101 MHz, CHLOROFORM-*d*) δ ppm 166.3, 159.6, 159.3, 152.9, 148.4, 130.3 (2 x CH), 130.2 (2 x CH), 129.7, 129.4 (2 x CH), 128.7, 128.1, 127.0, 123.8 (s), 122.9 (s), 118.2 (s), 113.9 (4 x CH), 113.5 (2 x CH), 75.2, 71.2, 66.7, 55.3, 55.3, 55.2

HRMS (ES+) $C_{31}H_{30}O_7Na$ $[M+ Na]^+$ requires 537.1889 found 537.1913

spectroscopic data consistent with literature[3]

2,3-Bis((4-methoxybenzyl)oxy)benzoic acid (**7**)

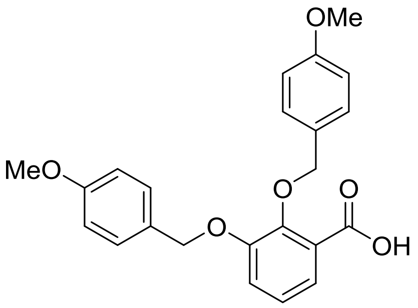

A solution of **6** (0.50 g, 0.97 mmol) in 1,4-dioxane (4.88 mL) and NaOH (aqueous, 2 M, 2.44 mL) was stirred vigorously at room temperature for 24 h. The reaction mixture was concentrated in vacuo to remove the organic solvent. The remaining residue was stirred in water (6 mL) and acidified to pH 2 with the dropwise addition of HCl (aqueous, 1 M). The white solid was filtered and washed with *n*-hexanes to yield compound **7** as a white solid (0.27 g, 0.69 mmol, 71%).

^{1}**H NMR** (400 MHz, CHLOROFORM-*d*) δ ppm 7.73 (1 H, dd, *J*=7.8, 1.8 Hz), 7.41 (2 H, d, *J*=8.6 Hz), 7.22 - 7.27 (3 H, m), 7.17 (1 H, t, *J*=8.0 Hz), 6.96 (2 H, d, *J*=8.6 Hz), 6.83 (2 H, d, *J*=8.6 Hz), 5.20 (2 H, s), 5.12 (2 H, s), 3.84 (3 H, s), 3.80 (3 H, s)

^{13}**C NMR** (101 MHz, CHLOROFORM-*d*) δ ppm 165.2, 160.4, 151.4, 147.1, 131.1 (2 x CH), 129.6 (2 x CH), 128.0, 126.8, 124.9, 124.3, 123.0, 119.1, 114.2 (4 x CH), 76.9, 71.4, 55.3, 55.3;

HRMS (ES+) $C_{23}H_{22}O_6Na$ $[M+Na]^+$ requires 417.1314 found 417.1324 (spectroscopic data consistent with literature).[3]

Methyl N2,N6-bis(2,3-bis((4-methoxybenzyl)oxy)benzoyl)-L-lysinate (**8**)

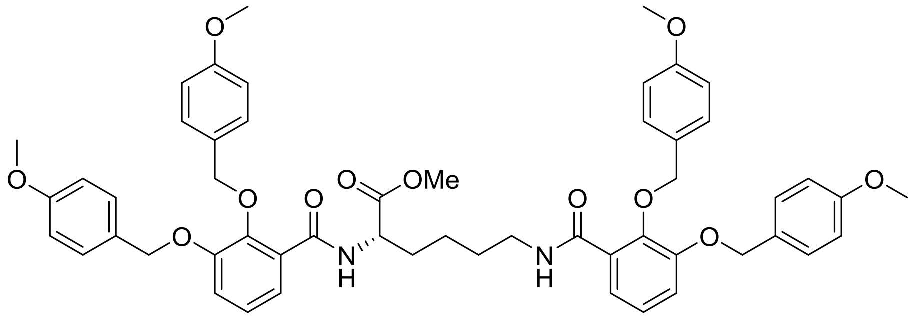

To **7** (5.00 g, 12.6 mmol), *N,N'*-dicyclohexylcarbodiimide (2.62 g, 12.6 mmol) and ethyl (hydroxyimino)cyanoacetate (1.80 g, 12.6 mmol) was added DMF (150 mL) and the reaction mixture left to stir for 11 min at 0 °C. Diisopropylethylamine (2.1 mL, 12.6 mmol) followed by L-lysine methyl ester dihydrochloride (1.47 g, 6.3 mmol) was then added to the reaction mixture. The orange reaction solution was then left to stir at room temperature for 22 h. EtOAc (150 mL) was added to the reaction mixture and cooled to 0 °C for 30 mins. The white precipitate formed (dicyclohexylurea) was decanted from the solution. The remaining organic solution was then washed with HCl (aqueous, 1 M, 2 × 200 mL), $NaHCO_3$ (10% aqueous solution, 2 × 200 mL) and LiCl (5 % aqueous solution, 2 × 150 mL). The organic phase was dried over anhydrous Na_2SO_4, filtered under gravity and the organic solvent removed in vacuo. The crude material (4.65 g) was dissolved in acetonitrile (50 mL) and left at 4 °C overnight to precipitate and remove further dicyclohexylurea. The white precipitate dicyclohexylurea was filtered and the acetonitrile removed in vacuo. The remaining material was purified by flash chromatography on silica (stepped gradient 50–70% EtOAc in petroleum ether (40–60)) to yield compound **8** as an off orange gum (3.20 g, 3.51 mmol, 56%).

^{1}H NMR (500 MHz, CHLOROFORM-*d*) δ ppm 8.45 (1 H, d, *J*=7.5 Hz), 7.86 (1 H, br t, *J*=5.2 Hz), 7.60 - 7.68 (2 H, m), 7.32 (4 H, d, *J*=8.1 Hz), 7.21 (2 H, d, *J*=8.5 Hz), 7.13 (2 H, d, *J*=8.1 Hz), 7.01 - 7.09 (4 H, m), 6.86 (4 H, d, *J*=8.3 Hz), 6.74 (2 H, d, *J*=8.5 Hz), 6.70 (2 H, d, *J*=8.5 Hz), 4.94 - 5.05 (6 H, app m), 4.90 (2 H, s), 4.55 (1 H, br q, *J*=5.8 Hz), 3.76 (6 H, s), 3.68 (6 H, s), 3.64 (3 H, s), 3.00 - 3.16 (2 H, m), 1.57 - 1.66 (1 H, m), 1.31 - 1.42 (1 H, m), 1.18 - 1.23 (2 H, m), 1.01 - 1.14 (2 H, m);

^{13}C NMR (101 MHz, CHLOROFORM-*d*) δ ppm 172.8 (C), 165.1 (2 x C), 159.9 (C), 159.8 (C), 159.7 (C), 159.7 (C) 151.8 (C), 151.8 (C), 147.1 (C), 146.9 (C), 130.6 (2 x CH), 130.5 (2 x CH), 129.6 (2 x CH), 129.5 (2 x CH), 128.6 (3 x C),128.5 (C) 127.3 (C), 126.5 (C), 124.2 (2 x CH), 123.2 (CH), 123.2 (CH) 117.3 (CH), 117.0 (CH), 114.1 (4 x CH), 114.0 (2 x CH), 113.8 (2 x CH), 76.0 (CH_2), 75.8 (CH_2), 71.1 (2 x CH_2), 55.3 (2 x CH_3), 55.2 (2 x CH_3), 52.6 (CH or CH_3), 52.2 (CH or CH_3), 39.3 (CH_2), 31.7 (CH_2), 28.9 (CH_2), 23.1 (CH_2)

HRMS (ES+) $C_{53}H_{57}N_2O_{12}$ $[M+H]^+$ requires 913.3912 found 913.3898

IR: *v* (cm^{-1}) (nujol) = 3369 (m, amide N-H), 2934 (s, CH), 1740 (s, CO) 1652 (s, CO)

$[\alpha]_D^{25}$ = -15.0 (c 1.00, $CHCl_3$)

N2,N6-Bis(2,3-bis((4-methoxybenzyl)oxy)benzoyl)-L-lysine (**9**)

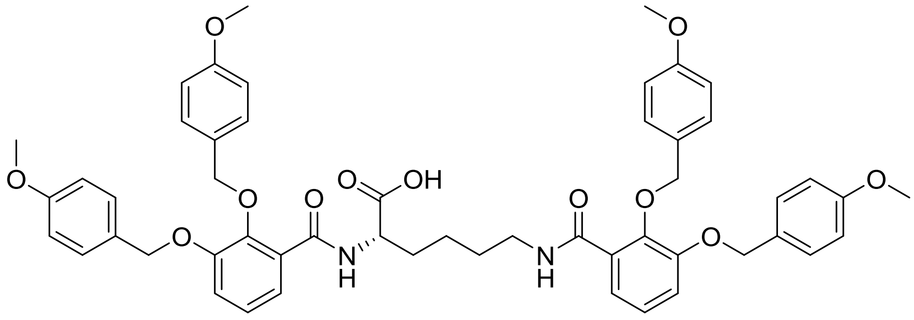

Compound **8** (0.42 g, 0.45 mmol) was dissolved in THF (20 mL) and LiOH (aqueous, 1 M, 4 mL) was added. The reaction was left to stir vigorously at room temperature until TLC indicated the reaction had gone to completion after 7 h. The reaction mixture was acidified to pH 2 with the dropwise addition of HCl (aqueous, 1 M). EtOAc (20 mL) was added and the phases were separated. The organic phase was collected and aqueous phase was extracted further with EtOAc (2 × 20 mL). The organic phases where combined, dried with anhydrous Na_2SO_4, filtered under gravity and the organic solvent removed in vacuo to yield compound **9** as an amorphous orange solid (0.39 g, 0.43 mmol, 96%).

^{1}H NMR (400 MHz, CHLOROFORM-*d*) δ ppm 8.62 (1 H, d, *J*=6.8 Hz), 8.00 (1 H, br t, *J*=5.6 Hz), 7.66 - 7.73 (2 H, m), 7.38 (4 H, d, *J*=8.6 Hz), 7.23 (2 H, d, *J*=8.6 Hz), 7.19 (2 H, d, *J*=8.8 Hz), 7.09 - 7.16 (4 H, m), 6.90 - 6.96 (4 H, m), 6.79 - 6.84 (2 H, m), 6.74 - 6.79 (2 H, m), 5.00 - 5.11 (6 H, app m), 4.97 (2 H, s), 4.49 (1 H, q, *J*=7.1 Hz), 3.83 (6 H, s), 3.75 (3

H, s), 3.72 (3 H, s), 3.13 - 3.21 (2 H, m), 1.69 - 1.79 (1 H, m), 1.37 - 1.48 (1 H + H_2O, m), 1.24 - 1.28 (2 H, m), 1.16 - 1.23 (2 H, m);

^{13}C NMR (101 MHz, CHLOROFORM-*d*) δ ppm 174.4 (C), 165.9 (C), 165.3 (C), 159.9 (C), 159.9 (C), 159.7 (C), 159.7 (C), 151.8 (C), 151.7 (C), 147.2 (C), 146.9 (C), 130.8 (2 x CH), 130.5 (2 x CH), 129.6 (2 x CH), 129.5 (2 x CH), 128.5 (C), 128.5 (C) 128.4 (C), 128.4 (C), 127.1 (C), 126.1 (C), 124.2 (2 x CH), 123.2 (CH), 123.2 (CH), 117.5 (CH), 117.1 (CH), 114.1 (4 x CH), 114.0 (2 x CH), 113.9 (2 x CH), 76.1 (CH_2), 76.0 (CH_2), 71.1 (CH_2), 71.1 (CH_2) 55.3 (2 x CH_3), 55.3 (CH_3), 55.2 (CH_3), 52.9 (CH), 39.4 (CH_2), 31.1 (CH_2), 28.9 (CH_2), 23.0 (CH_2)

HRMS (ES+) $C_{52}H_{55}N_2O_{12}$ $[M+ H]^+$ requires 899.3755 found 899.3746

IR: *v* (cm^{-1}) (neat) = 3364 (m, amide N-H), 2934 (br, CH and OH), 1734 (s, CO) 1611(s, CO)

$[\alpha]_D^{25}$ = -21.6 (c 1.16, $CHCl_3$)

N-(4-(10-(2,3-Bis((4-methoxybenzyl)oxy)benzamido)-16-(2,3-bis((4-methoxyphenoxy)methyl)phenyl)-9,16-dioxo-2,5-dioxa-8,15-diazahexadecyl)benzyl)-3-(2-chloro-10H-phenothiazin-10-yl)-N,N-dimethylpropan-1-aminiun trifluoroacetate (**10**)

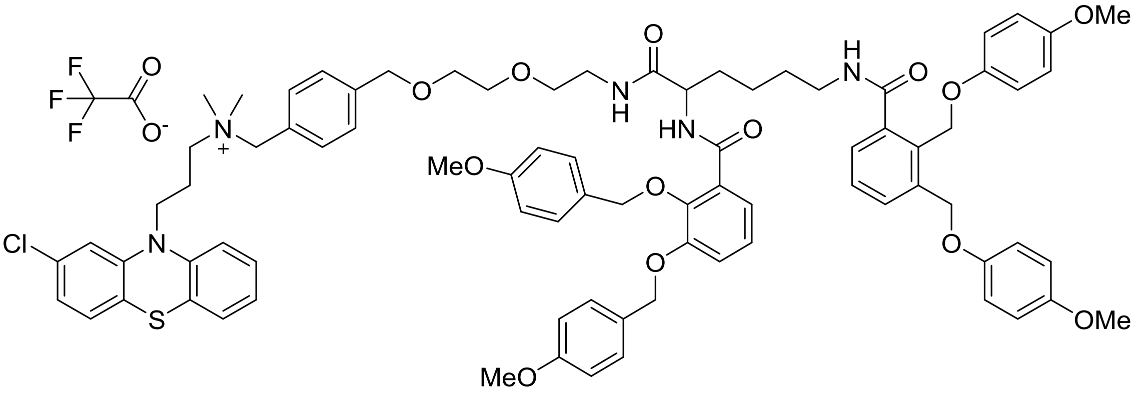

To acid **9** (0.15 g, 0.17 mmol) was added HATU (0.06 g, 0.15 mmol) and DMF (1.5 mL) followed by DIPEA (0.06 ml, 0.34 mmol) and the reaction mixture was left to stir at room temperature for 20 min. The amine **5** (0.16 g, 2.16 mmol) and DIPEA (0.03 mL, 0.17 mmol) in DMF (1.5 mL) was added to the reaction mixture. The reaction was left to stir at room temperature for 18 h. The DMF was removed in vacuo and the remaining redidue was

purififed by flash chromatography on silica (stepped gradient 3–5 % methanol in DCM) to yield compound **10** as a white solid (0.07 g, 0.05 mmol, 33%).

^{1}H NMR (500 MHz, CHLOROFORM-*d*) δ ppm 8.45 (1 H, d, *J*=8.5 Hz), 7.94 (1 H, br t, *J*=5.4 Hz), 7.58 - 7.63 (2 H, m), 7.34 - 7.40 (4 H, m), 7.26 (5 H, t, *J*=7.0 Hz), 7.18 (5 H, t, *J*=7.0 Hz), 7.06 - 7.15 (6 H, m), 7.00 (1 H, d, *J*=8.1 Hz), 6.92 (5 H, dd, *J*=8.7, 2.4 Hz), 6.79 (2 H, d, *J*=8.7 Hz), 6.76 (2 H, d, *J*=8.7 Hz), 6.71 (1 H, t, *J*=5.8 Hz), 5.00 - 5.05 (6 H, app m), 4.95 (2 H, s), 4.49 (2 H, s), 4.38 (1 H, q, *J*=6.6 Hz), 4.25 (2 H, s), 3.95 (2 H, app br s), 3.81 (6 H, s), 3.73 (3 H, s), 3.71 (3 H, s), 3.59 - 3.62 (2 H, m), 3.56 - 3.58 (2 H, m), 3.54 (2 H, br t, *J*=5.6 Hz), 3.41 - 3.47 (2 H, m), 3.29 - 3.33 (2 H, m), 3.11 - 3.18 (2 H, m), 2.82 (6 H, s), 2.22 (2H, app b s), 1.64 - 1.72 (1 H, m), 1.34 - 1.43 (1 H, m), 1.22 - 1.29 (2 H, m), 1.13 - 1.19 (2 H, m)

^{13}C NMR (126 MHz, CHLOROFORM-*d*) δ ppm 171.6 (C), 165.3 (C), 165.1 (C), 159.9 (C), 159.8 (C), 159.7 (C), 159.7 (C), 151.8 (C), 151.8 (C), 146.9 (C), 146.8 (C), 146.2 (C), 143.3 (C), 141.6 (C) 133.7 (C), 132.7 (2 x CH), 130.8 (2 x CH), 130.5 (2 x CH), 129.7 (2 x CH), 129.7 (2 x CH), 129.5 (2 x CH), 128.5 (C), 128.5 (C), 128.3 (C), 128.2 (CH), 128.2 (CH), 128.1 (CH), 127.8 (C), 127.2 (C), 126.5 (C), 125.7 (C), 124.4 (C), 124.4 (C), 124.3 (CH), 124.3 (CH), 124.3 (CH), 123.8 (CH), 123.1 (CH), 122.9 (CH), 122.8 (CH), 117.4 (CH), 117.0 (CH), 116.7 (CH), 116.4 (CH), 114.1 (4 x CH), 114.0 (2 x CH), 113.9 (2 x CH), 76.0 (CH_2), 75.7 (CH_2), 72.2 (CH_2), 71.1 (CH_2), 71.0 (CH_2), 70.3 (CH_2), 69.9 (CH_2), 69.7 (CH_2), 68.4 (CH_2), 61.6 (CH_2),55.3 (2 x CH_3), 55.3 (CH_3), 55.2 (CH_3), 53.7 (CH), 49.8 (2 x CH_3), 43.7 (CH_2), 39.3 (2 x CH_2), 31.5 (CH_2), 29.0 (CH_2), 23.2 (CH_2), 20.2 (CH_2)

HRMS (ES+) $C_{81}H_{89}N_5O_{13}S$ ^{35}Cl $[M]^+$ requires 1406.5866 found 1406.5823; $C_{81}H_{89}N_5O_{13}S$ ^{37}Cl $[M]^+$ requires 1408.5837 found 1408.5847

IR: ν (cm^{-1}) (neat) = 3651 (m, amide N-H), 2980, 2887 (s, CH), 1645 (s, CO).

3-(2-Chloro-10H-phenothiazin-10-yl)-N-(4-(10-(2,3-dihydroxybenzamido)-16-(2,3-dihydroxyphenyl)-9,16-dioxo-2,5-dioxa-8,15-diazahexadecyl)benzyl)-N,N-dimethylpropan-1-aminium trifluoroacetate (**11**)

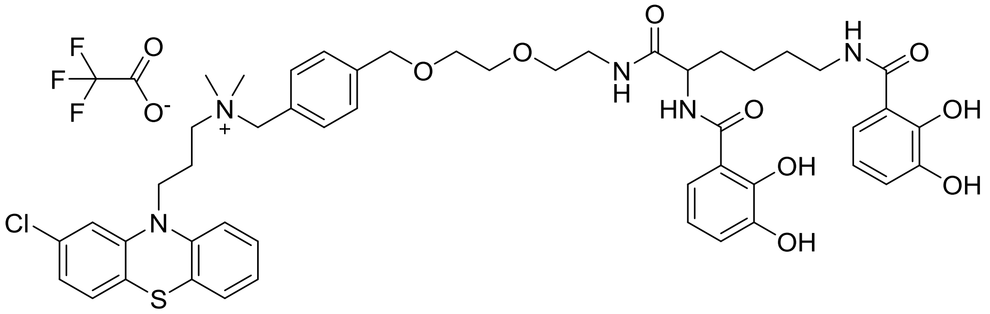

To **10** (30.0 mg, 1.95 × 10^{-5} mol) was added DCM (0.2 mL), anisole (50.0 µL, 0.46 mmol) and TFA (0.2 mL, 2.6 mmol) at 0 °C. The pink reaction mixture was left to stir at 0 °C for 1 h followed by stirring at room temperature for 1 h. The solvent was removed in vacuo and azeotroped with DCM. The residue was then triturated with diethyl ether (3 × 1 mL). The reaming crude product (25.0 mg) was purified by semi-preparative HPLC, acetonitrile water gradient 0.1% TFA buffer, (Phenomenex Luna Omega 1.6 µm Polar C18 stationary phase) and lyophilized to yield compound 11 (10.8 mg, 1.02 × 10^{-5} mol, 53%) as a white fluffy powder.

^{1}H NMR (400 MHz, METHANOL-d_4) δ ppm 7.25 (2 H, d, *J*=7.8 Hz), 7.11 - 7.22 (4 H, m), 7.04 - 7.09 (2 H, m), 7.00 (1 H, d, *J*=8.1 Hz), 6.92 - 6.97 (2 H, m), 6.89 (2 H, td, *J*=8.3, 2.0 Hz), 6.82 (2 H, td, *J*=7.6, 1.2 Hz), 6.56 - 6.64 (2 H, m), 4.42 - 4.50 (3 H, m), 4.27 (2 H, s), 3.99 (2 H, t, *J*=5.9 Hz), 3.56 (4 H, app s), 3.48 (2 H, t, *J*=5.9 Hz), 3.29 - 3.38 (2 H, m), 3.22 - 3.28 (4 H, m), 2.82 (6 H, s), 2.11 - 2.22 (2 H, m), 1.78 - 1.87 (1 H, m), 1.67 - 1.77 (1 H, m), 1.54 (2 H, quin, *J*=7.1 Hz), 1.31 - 1.42 (2 H, m);

^{13}C NMR (101 MHz, METHANOL-d_4) δ ppm 173.0 (C), 170.1 (C), 169.3 (C), 148.8 (C), 148.2 (C), 146.4 (C), 145.9 (C), 145.8 (C), 143.8 (C), 141.4 (C), 133.4 (C), 132.4 (2 x CH), 128.0 (CH), 127.9 (2 x CH), 127.6 (CH), 127.4 (CH), 126.1 (C), 125.8 (C), 124.7 (C), 123.4 (CH), 122.7 (CH), 118.5 (CH), 118.4 (CH), 118.2 (3 x CH), 117.2 (CH), 116.4 (CH), 116.1 (CH), 115.8 (C), 115.4 (C), 71.9 (CH_2), 70.0 (CH_2), 69.8 (CH_2), 69.1 (CH_2), 67.9 (CH_2), 61.3 (CH_2), 53.5 (CH or 2 x CH_3), 49.2 (CH or 2 x CH_3), 43.7 (CH_2), 39.1 (CH_2), 38.8 (CH_2), 31.6 (CH_2), 28.7 (CH_2), 22.9 (CH_2), 20.1 (CH_2);

HRMS (ES+) $C_{49}H_{57}N_5O_9S$ ^{35}Cl $[M]^+$ requires 926.3566 found 926.3572; $C_{49}H_{57}N_5O_9S$ ^{37}Cl $[M]^+$ requires 928.3536 found 928.3567

IR: ν (cm^{-1}) (neat) = 3659 (m, amide N-H), 3352 (br, OH), 2980, 2887 (s, CH), 1639 (s, CO).

Biological evaluation

Determination of Minimum Inhibitory Concentration (MIC) was undertaken as described in literature,[4] but using Sauton's medium devoid of iron and zinc with 0.01% w/v resazurin.[5] Compound **4**, pre-dissolved in dimethyl sulphoxide (DMSO) to a concentration of 200 mM, was diluted to 50 μM in the first row and serially 2-fold diluted into subsequent rows, with the final row kept devoid of the compound as a positive control for mycobacterial growth. Mycobacterial growth was not affected by the concentrations of DMSO used in this study.

Overnight subcultures of *Mycobacterium smegmatis* MC^{2}155 were diluted to optical density 0.1 and 5 μL of this suspension used to inoculate three of four columns. Plates were read after 48 hours static incubation at 37 °C using colour change from resazurin oxidation to indicate growth; as was observed at 6.25 μM and above in each inoculated column. Results remained unchanged after further incubation of up to 7 days. Bacterial inoculum was confirmed, by Colony Forming Unit (CFU) counts on 7H10 Middlebrook agar, as ~1 × 10^5 CFU/mL.

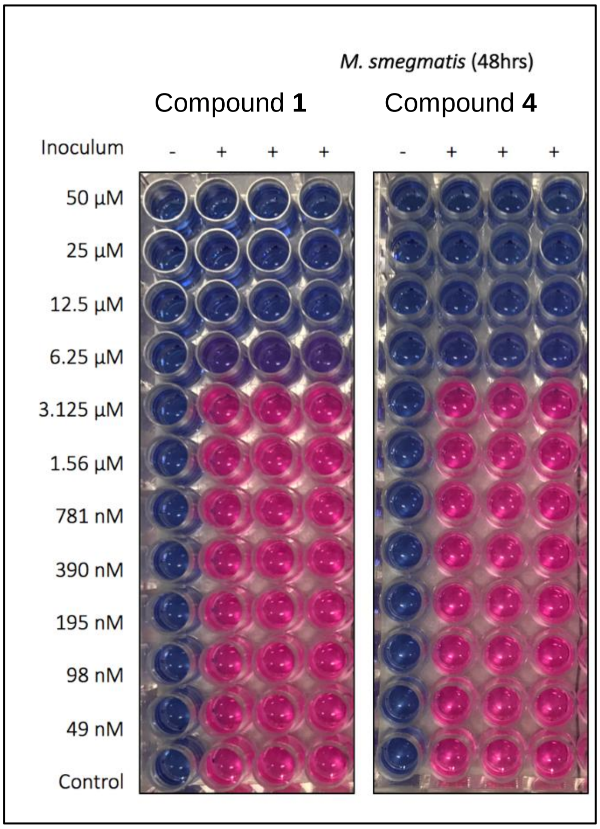

References

1. Khan, M.O.F.; Austin S.E.; Chan, C.; Yin, H.; Marks, D.;Vaghjiani, S.N.; Kendrick, H.;Yardley, V.; Croft, S.L.; Douglas, K.T. *J. Med. Chem.*, **2000**, *43*, 3148–3156.
2. Zhang, X.X.;Prata, C.A.H.; McIntosh, T.J.; Barthélémy, P.; Grinstaff. M.W. *Bioconjugate Chem.* **2010**, *21*, 988–993.
3. Bergeron, R.J.;Bharti, N.; Singh, S.; McManis, J.S.;Wiegand, J.; Green, L.G.; *J Med Chem.* **2009**, *52*, 3801–3813.
4. Turapov, O.; Loraine, J.; Jenkins, C.H.; Barthe, P.; McFeely, D.; Forti, F.; Ghisotti, D.; Hesek, D.; Lee, M.; Bottrill, A.R.; Vollmer, W.; Mobashery, S.; Cohen-Gonsaud M.; Mukamolova, G.V. **2015** *Open Biology*, *5*, 150025.
5. Connell, N. D. **1995** *Methods in Cell Biology*, *45*, 107–125.

Novel spectra

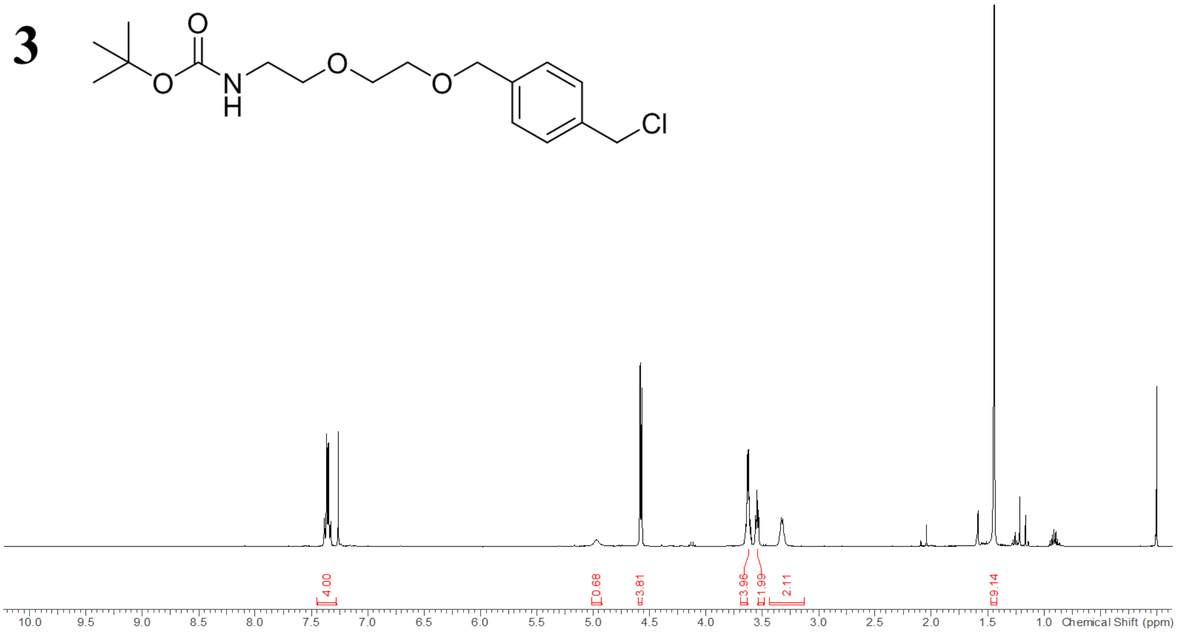

^{1}H NMR spectrum of compound 3 in $CDCl_3$

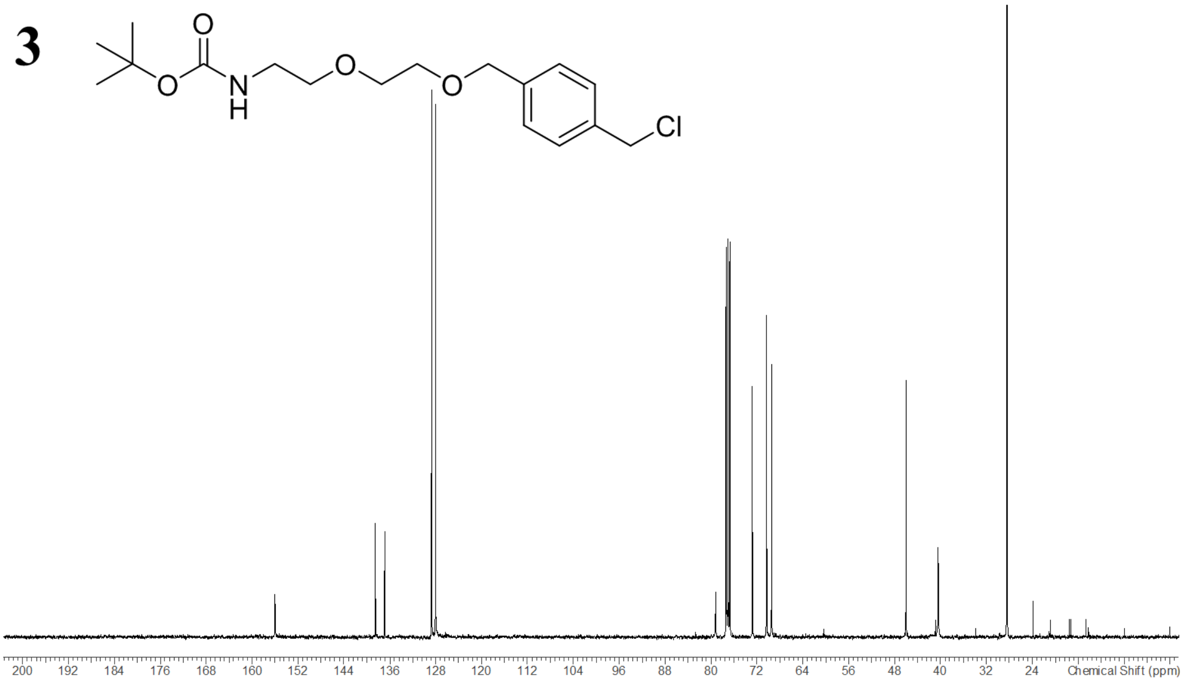

^{13}C NMR spectrum of compound 3 in $CDCl_3$

4

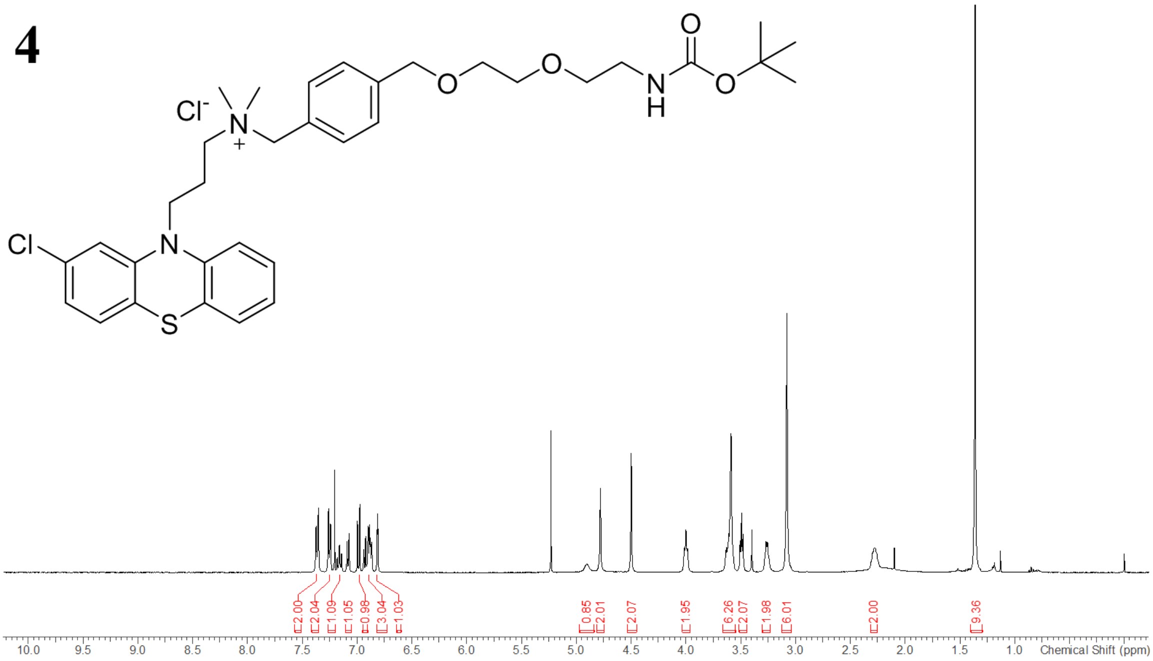

^{1}H NMR spectrum of compound 4 in $CDCl_3$

4

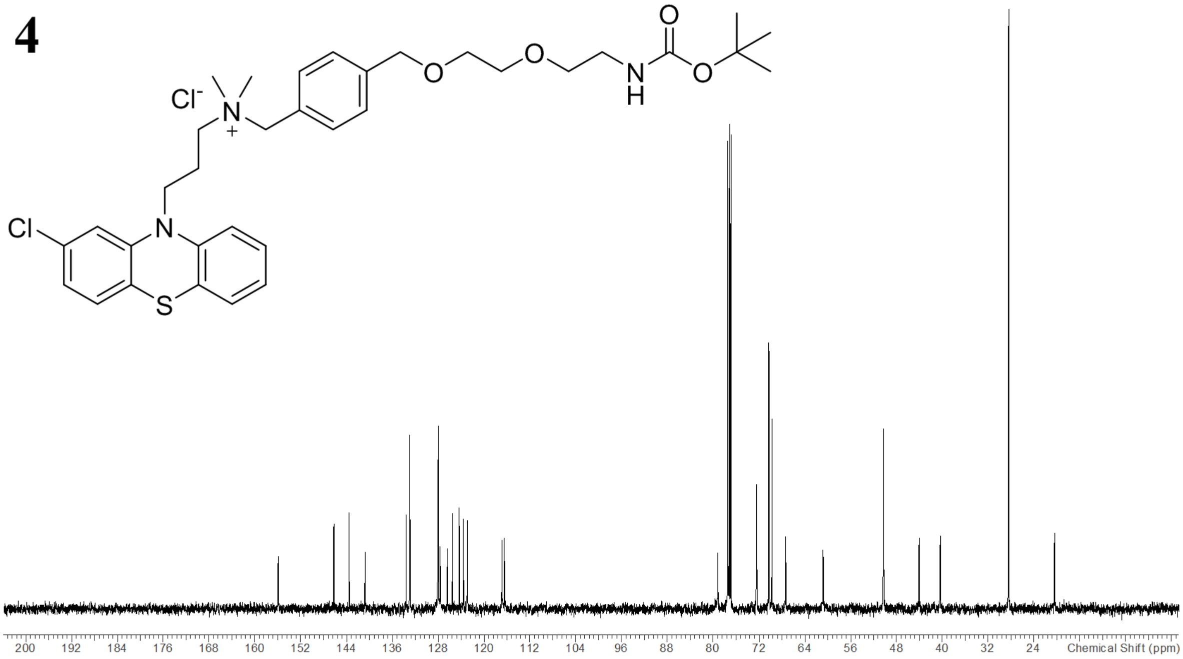

^{13}C NMR spectrum of compound 4 in $CDCl_3$

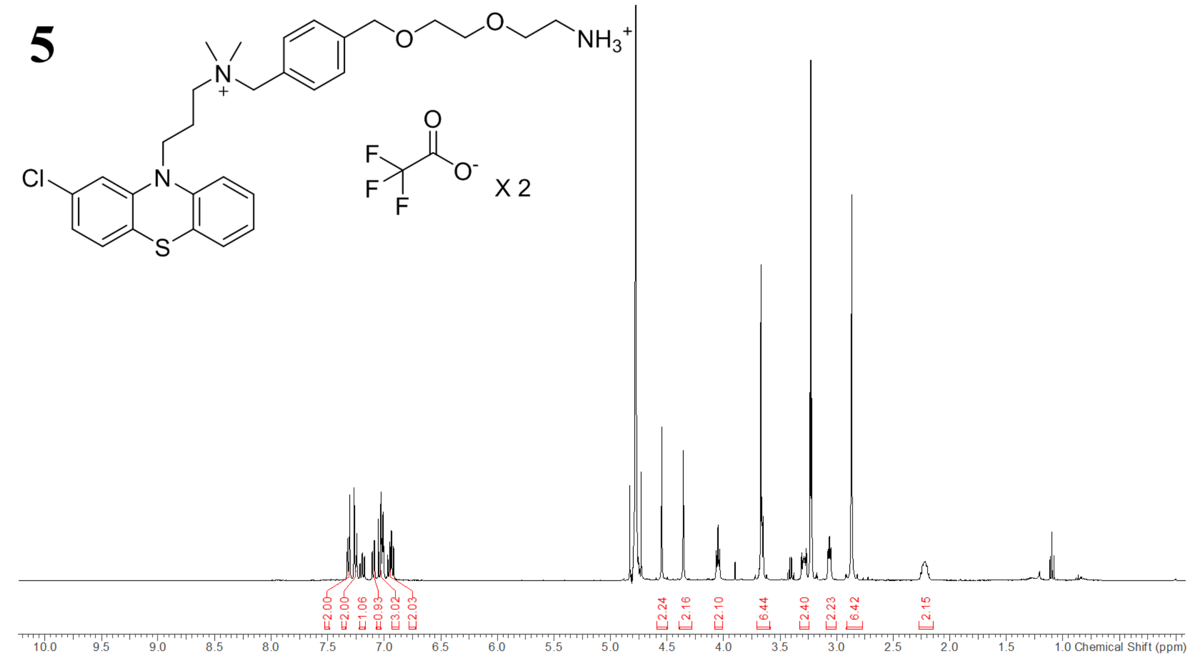

^{1}H NMR spectrum of compound 5 in CD_3OD

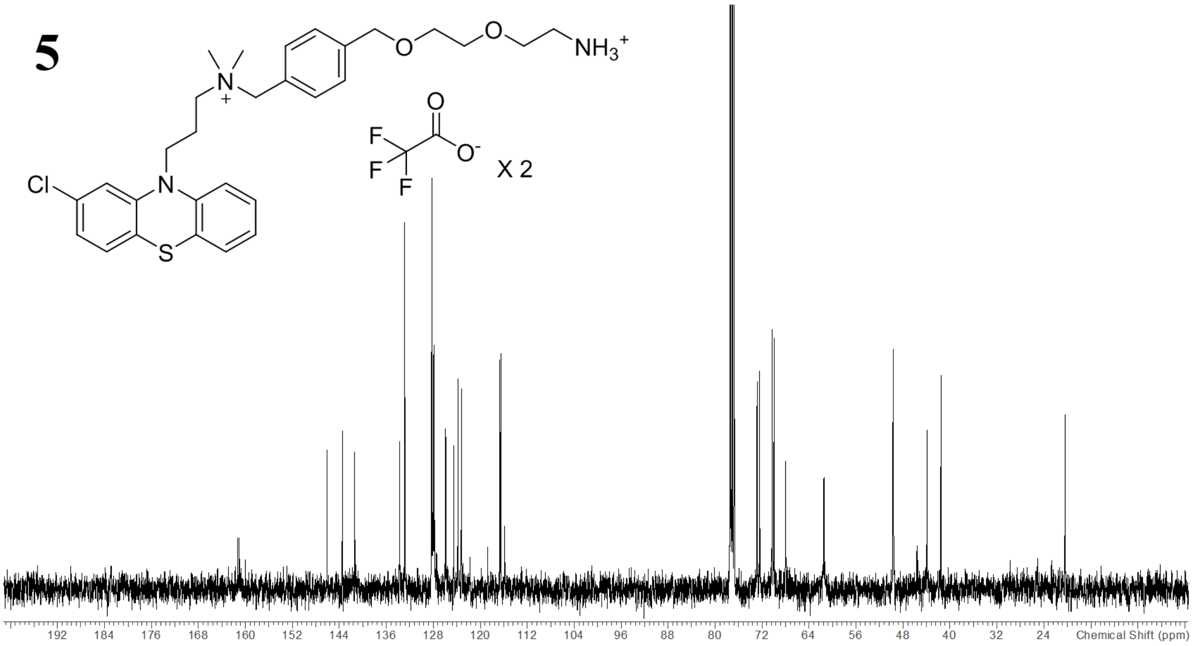

^{13}C NMR spectrum of compound 5 in $CDCl_3$

8

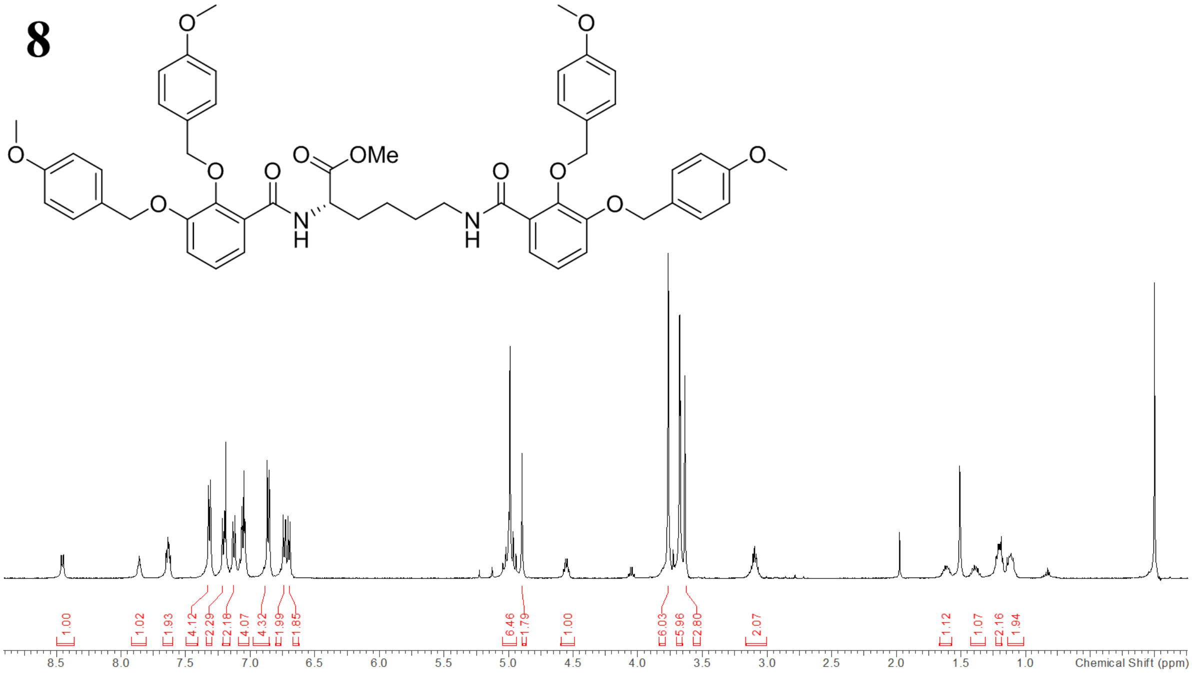

^{1}H NMR spectrum of compound 8 in $CDCl_3$

8

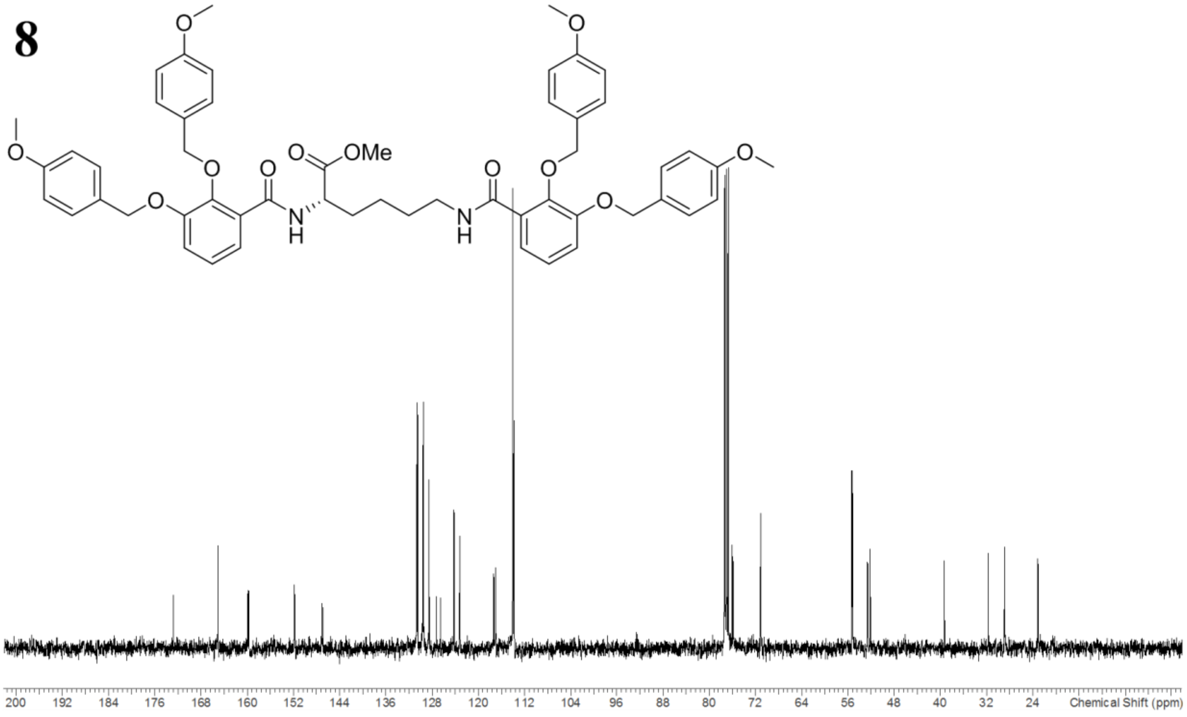

^{13}C NMR spectrum of compound 8 in $CDCl_3$

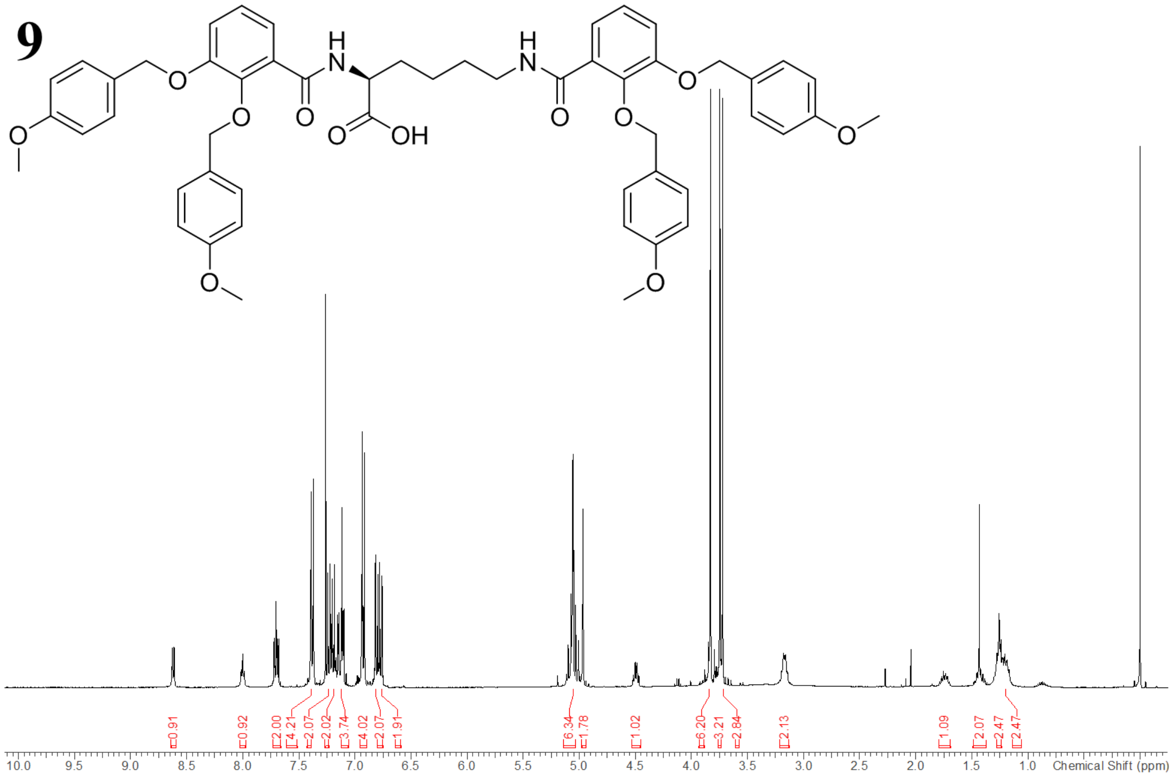

^{1}H NMR spectrum of compound 9 in $CDCl_3$

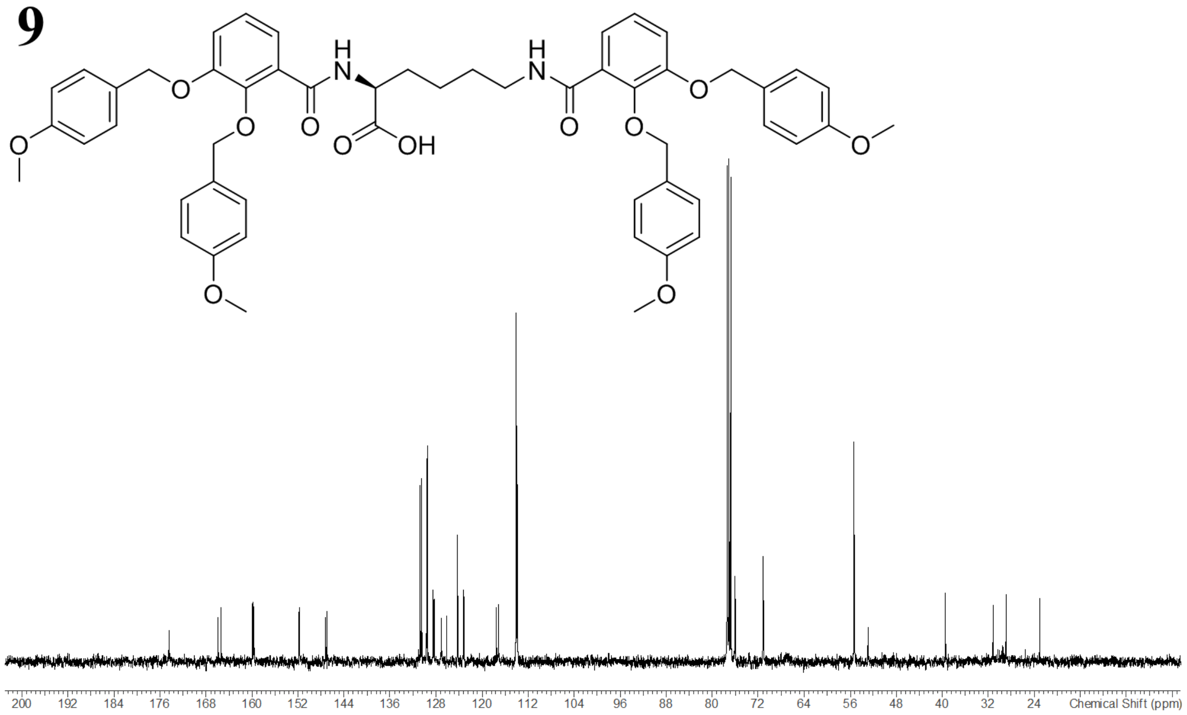

^{13}C NMR spectrum of compound 9 in $CDCl_3$

10

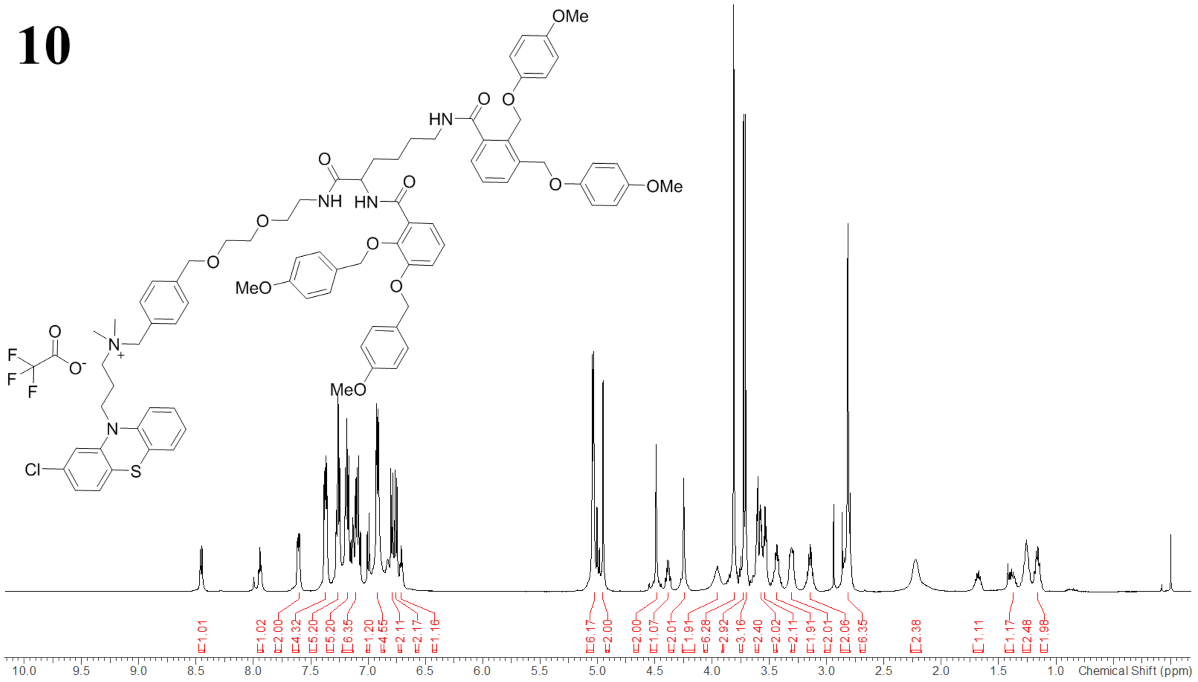

^1H NMR spectrum of compound 10 in $CDCl_3$

10

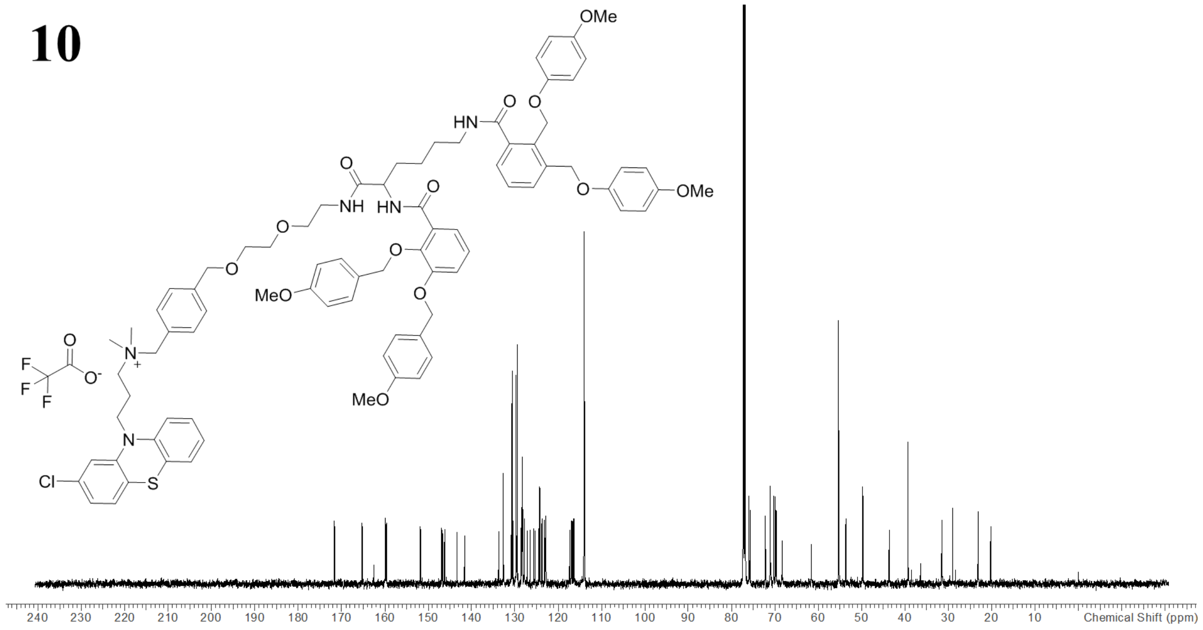

^{13}C NMR spectrum of compound 10 in $CDCl_3$

11

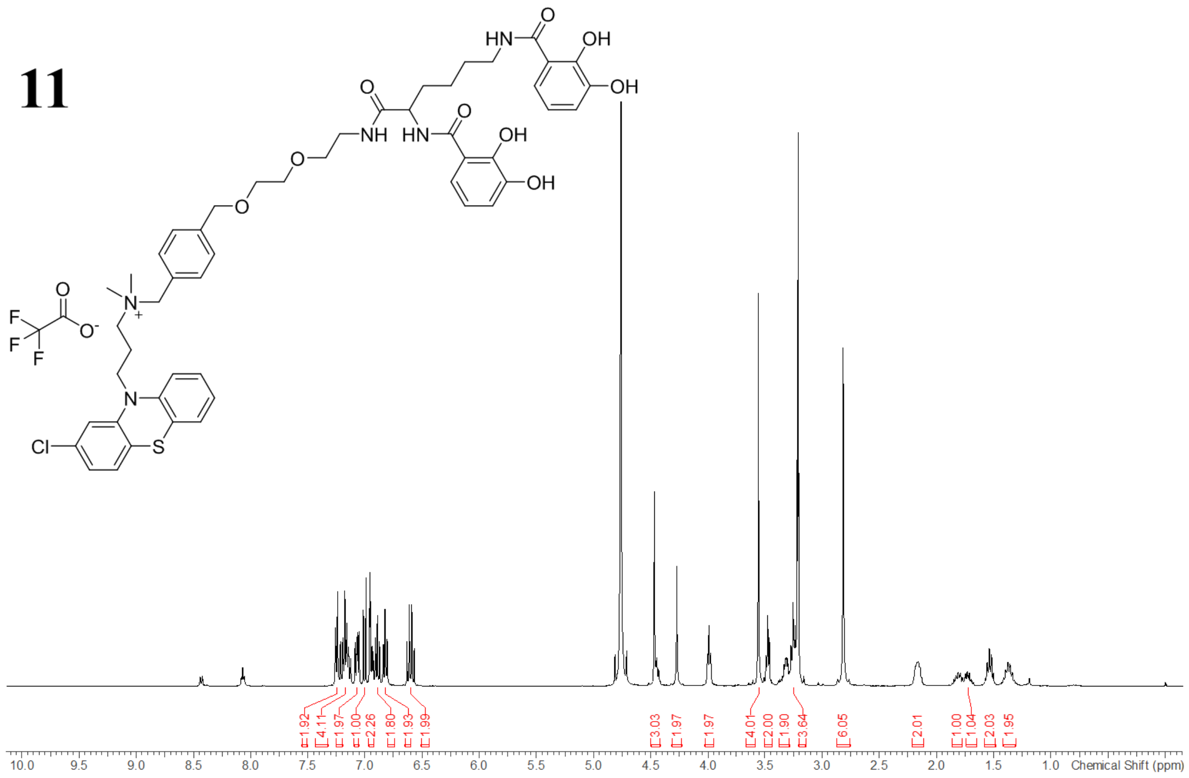

^{1}H NMR spectrum of compound 11 in CD_3OD

11

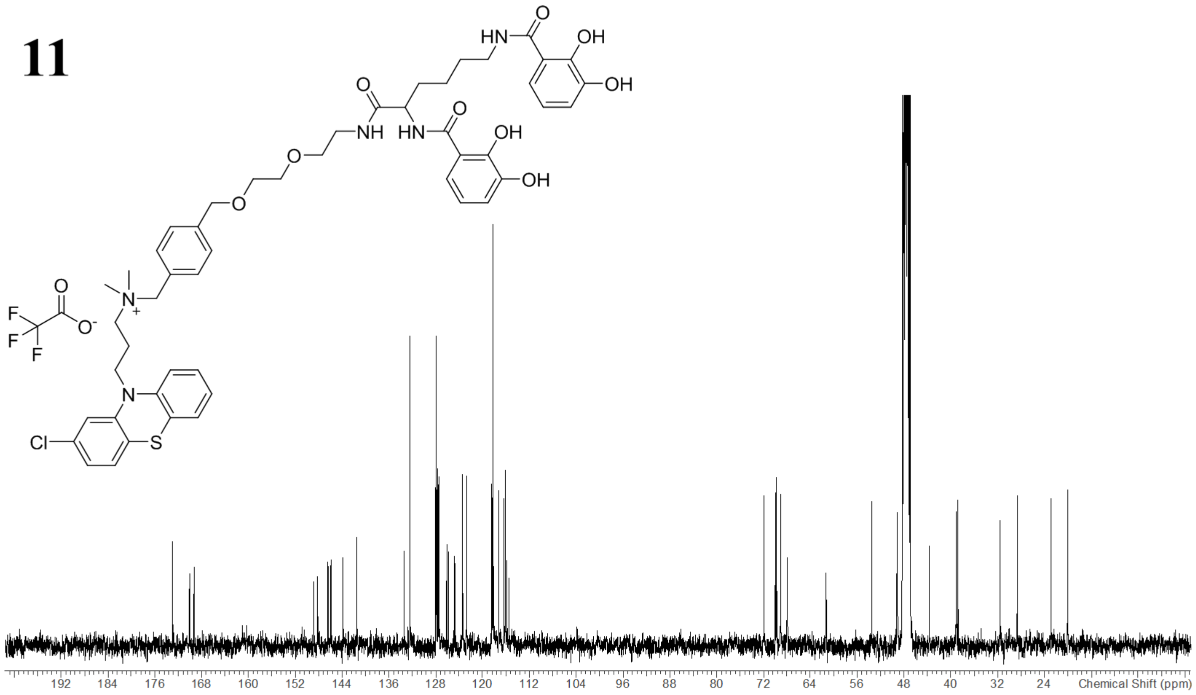

^{13}C NMR spectrum of compound 11 in CD_3OD
